# A Pilot Investigation of Visceral Fat Adiposity and Gene Expression Profile in Peripheral Blood Cells

**DOI:** 10.1371/journal.pone.0047377

**Published:** 2012-10-16

**Authors:** Masaya Yamaoka, Norikazu Maeda, Seiji Nakamura, Susumu Kashine, Yasuhiko Nakagawa, Aki Hiuge-Shimizu, Kohei Okita, Akihisa Imagawa, Yuji Matsuzawa, Ken-ichi Matsubara, Tohru Funahashi, Iichiro Shimomura

**Affiliations:** 1 Department of Metabolic Medicine, Graduate School of Medicine, Osaka University, Suita, Osaka, Japan; 2 DNA Chip Research Inc., Yokohama, Kanagawa, Japan; 3 Sumitomo Hospital, Osaka, Osaka, Japan; University of Sao Paulo, Brazil

## Abstract

Evidence suggests that visceral fat accumulation plays a central role in the development of metabolic syndrome. Excess visceral fat causes local chronic low-grade inflammation and dysregulation of adipocytokines, which contribute in the pathogenesis of the metabolic syndrome. These changes may affect the gene expression in peripheral blood cells. This study for the first time examined the association between visceral fat adiposity and gene expression profile in peripheral blood cells. The gene expression profile was analyzed in peripheral blood cells from 28 obese subjects by microarray analysis. Reverse transcription-polymerase chain reaction (RT-PCR) was performed using peripheral blood cells from 57 obese subjects. Obesity was defined as body mass index (BMI) greater than 25 kg/m^2^ according to the Japanese criteria, and the estimated visceral fat area (eVFA) was measured by abdominal bioelectrical impedance. Analysis of gene expression profile was carried out with Agilent whole human genome 4×44 K oligo-DNA microarray. The expression of several genes related to circadian rhythm, inflammation, and oxidative stress correlated significantly with visceral fat accumulation. Period homolog 1 (PER1) mRNA level in blood cells correlated negatively with visceral fat adiposity. Stepwise multiple regression analysis identified eVFA as a significant determinant of PER1 expression. In conclusion, visceral fat adiposity correlated with the expression of genes related to circadian rhythm and inflammation in peripheral blood cells.

## Introduction

It has been shown that there is a significant association between computed tomography (CT)-based fat distribution and life style-related diseases, such as diabetes, dyslipidemia, and hypertension. Visceral fat-related obesity is closely associated with the development of atherosclerotic diseases [1]. The metabolic syndrome is strongly linked to visceral fat adiposity. The exact pathomechanisms of the metabolic syndrome are not clear at present but seem to involve accumulation of macrophages in adipose tissue, which induce a state of chronic low-grade inflammation by producing a battery of inflammatory mediators. In addition, these macrophages interact with adipocytes through free fatty acids and adipocytokines, creating a vicious cycle that promotes the development of the metabolic syndrome and atherosclerosis [2]–[4]. However, to date, there is no method to evaluate the function and condition of human visceral fat.

A series of recent studies demonstrated that the adipose tissue of obese subjects contains not only macrophages but also non-macrophage immunocytes, such as T-cells [5], [6], B-cells [7], and eosinophils [8], and that these cells accelerate the development of metabolic syndrome. These evidences imply that gene expression profile in peripheral blood cells may reflect the visceral fat condition. However, there is no report demonstrating the relation of peripheral blood gene expressions and visceral fat accumulation. Hence, the present study tested the association between visceral fat adiposity and the gene expression profile in peripheral blood cells to search novel surrogate markers relating to visceral fat adiposity and to establish novel diagnostic tools for metabolic syndrome.

## Materials and Methods

### Study Population

All subjects were inpatients of the Division of Endocrinology & Metabolism, Osaka University Hospital, Osaka. Written informed consent was obtained from each subject after explaining the purpose and potential complications of the study. The study protocol was approved by the human ethics committee of Osaka University and the study was registered with the University hospital Medical Information Network (Number: UMIN 000001663). Obesity was defined as body mass index (BMI) greater than 25 kg/m^2^ [9]. Subjects with type 1 diabetes mellitus, autoimmune diseases, malignant diseases, and infectious diseases were excluded from the study. Patients treated with statins and/or thiazolidinediones were also excluded. Sixty-two subjects were enrolled in the study, although five subjects were later excluded due to RNA degradation in the blood samples collected from these individuals. Thus, the present study was conducted in 57 obese patients.

### Clinical Parameters

The estimated visceral fat area (eVFA) was measured by abdominal bioelectrical impedance analysis (BIA), as reported previously [10], [11]. Physical examination and collection of blood samples were conducted on the same day. The homeostasis model−assessment of insulin resistance (HOMA-IR) was calculated by the equation: HOMA-IR = fasting insulin (µU/mL) x fasting glucose (mg/dL)/405.

The intima-media thickness (IMT) of the carotid arteries was measured using a high-resolution B-mode ultrasonography system (Xario; Toshiba Medical Systems Corp., Tochigi, Japan) with an electrical linear transducer (mid-frequency 7.5 MHz). IMT represented the distance between two parallel echogenic lines corresponding to the blood-intima and media-adventitia interfaces on the posterior wall of the artery. Three determinations of IMT were conducted at the site of the thickest point, maximum IMT (max-IMT) and two adjacent points (located 1 cm upstream and 1 cm downstream from this site). These three determinations were averaged and expressed as the mean IMT.

Type 2 diabetes mellitus was defined as fasting plasma glucose (FPG) concentration ≥126 mg/dL, 2-h plasma glucose concentration following 75 g oral glucose load of ≥200 mg/dL, or treatment with glucose-lowering agents. Hypertension was defined as systolic blood pressure (BP) ≥140 mmHg, diastolic BP≥90 mmHg, or treatment with anti-hypertensive agents. Dyslipidemia was defined as fasting triglycerides (TG) ≥150 mg/dL, high-density lipoprotein cholesterol (HDL-C) <40 mg/dL, or low-density lipoprotein cholesterol (LDL-C) ≥140 mg/dL, or treatment with lipid-lowering agents.

### Isolation of RNA

For total RNA isolation, blood samples were collected into PaxGene Blood RNA tubes (PreAnalytiX/QIAGEN Inc., Valencia, CA) at 7∶30 am and left to stand for 2 h at room temperature. The blood samples in the PaxGene Blood RNA tubes were stored at −20°C for 2 days and subsequently kept at −80°C until analysis. Total RNA was extracted by using PaxGene Blood RNA Kit (PreAnalytiX/QIAGEN) according to the protocol supplied by the manufacturer.

### Microarray Analysis

After RNA was qualified by the Agilent 2100 Bioanalyzer, 250 ng of total RNA was converted to cDNA, amplified, and labeled with Cy3-labeled CTP using the Quick Amp Labeling kit (Agilent Technologies, Santa Clara, CA) according to the protocol supplied by the manufacturer. Following labeling and clean up, the amplified RNA and dye incorporation were quantified using a ND-1000 Spectrophotometer (Nano Drop Technologies, Wilmington, DE) and hybridized to Agilent whole human genome 4 × 44 K oligo-DNA microarray (Agilent Technologies, Santa Clara, CA). After hybridization, the arrays were washed consecutively by using Gene Expression Wash Pack (Agilent Technologies). Fluorescence images of the hybridized arrays were generated using the Agilent DNA Microarray Scanner, and the intensities were extracted with Agilent Feature Extraction software ver.10.7.3.1. The raw microarray data are deposited in the National Center for Biotechnology Information Gene Expression Omnibus (GEO Series GSE28038).

### Real-Time RT-PCR

First-strand cDNA was synthesized from 180 ng of total RNA using Thermoscript RT (Invitrogen, Carlsbad, CA) and oligo dT primer. Real-time quantitative PCR amplification was conducted with the LightCycler 1.5 (Roche Diagnostics, Tokyo, Japan) using LightCycler-FastStart DNA Master SYBR Green I (Roche Diagnostics, Tokyo, Japan) according to the protocol recommended by the manufacturer. The final result for each sample was normalized to the respective GAPDH (glyceraldedyde-3-phosphate dehydrogenase) value. The primer sets used were: PER1, 5′-GAACTCAGATGTGGCTAGACC-3′ and 5′-TGTCAGCAACTTTGTCCAGGG-3′; GAPDH, 5′-AAGGGCATCCTGGGCTACA-3′ and 5′-GAGGAGTGGGTGTCGCTGTTG-3′.

### Microarray Data Analyses

The raw microarray intensities were processed by the percentile shift method (75th percentile) using the GeneSpring GX11 (Agilent Technologies) so as to normalize the range of expression intensities for inter-microarray. Only those genes whose expression data were available in more than 50% of hybridizations were included for further analyses. The normalized data were exported from the GeneSpring GX software. The correlation between peripheral blood gene expression levels and Log-eVFA levels was examined by Pearson’s correlation under the R environment (http://cran.at.r-project.org). Gene Ontology (GO) information was retrieved from the annotations in GeneSpring GX11.

### Clinical Data Analysis

Geometric mean values were used for insulin and C-reactive protein (CRP) due to the skewed distribution of the data. Non-normally distributed variables were log-transformed before analysis. The Spearman rank correlation coefficients for the study population as a whole were analyzed for Log-eVFA levels and other clinical variables. A P values less than 0.05 denoted the presence of significant difference. Pearson’s correlation coefficient was used to examine the relationship between period homolog 1 (PER1) and metabolic parameters. Stepwise multiple regression analysis with backward stepwise elimination was conducted to identify those parameters that significantly contributed to PER1. Log-eVFA, HOMA-IR, WBC and CRP were entered as independent variables in the analysis. All calculations were performed using the JMP software (JMP 9.0; SAS Institute Inc., Cary, NC). Data are expressed as mean±SD.

## Results

### Characteristics of the Subjects

The clinical characteristics of the participating subjects are listed in the Table 1. The mean BMI and eVFA of 57 patients were 30.6 kg/m^2^ (range, 25.4–51.2 kg/m^2^) and 166.8 cm^2^ (range, 80–386 cm^2^), respectively. The mean HOMA-IR was 3.0, reflecting mild insulin resistance. The proportion of patients with diabetes mellitus, dyslipidemia, and hypertension was 75%, 73%, and 57%, respectively. Frequency of patients treated with lipid-lowering drugs, anti-hypertensive drugs, oral glucose-lowering agents, insulin, and sleeping drugs was 25%, 42%, 18%, 42%, and 28%, respectively.

**Table 1:** Characteristics of participants.

N	57
Age (years)	51.7±13.5
Male/Female	27.0/30
Body weight (kg)	79.4±16.7
BMI (kg/m^2^)	30.6±5.3
Waist circumference (cm)	100.7±12.5
eVFA (cm^2^)	166.8±164.4
Log-eVFA	2.2±0.15
Systolic blood pressure (mmHg)	128.8±14.9
Diastolic blood pressure (mmHg)	76.3±10.9
Fasting glucose (mg/dL)	139.6±50.3
Hemoglobin A1c (%)	8.1±2.2
Immunoreactive insulin (µU/ml)	12.1±5.8
HOMA-IR (unit)	3.0±1.3
Total cholesterol (mg/dL)	210.4±37.1
LDL-C (mg/dL)	134.1±33.3
HDL-C (mg/dL)	46.8±10.2
Triglyceride (mg/dL)	155.7±76.8
Creatinine (mg/dL)	0.8±0.29
Ureic acid (mg/dL)	6.2±1.5
Serum adiponectin (µg/mL)	6.7±4
WBC (/µL)	6758.0±1838
Neutrophils (%)	55.2±7.5
Lymphocytes (%)	35.8±8
Eosinophils (%)	3.0±1.5
Basophils (%)	0.6±0.9
Monocytes (%)	7.4±7.5
RBC (×10^4^/µL)	466.0±53
Platelet (×10^4^/µL)	23.4±5.6
CRP (mg/dL)	0.4±0.48
Diabetes mellitus, n (%)	43 (75)
Dyslipidemia, n (%)	42 (73)
Hypertention, n (%)	33 (57)
Mean IMT (mm)	0.9±0.25
Medication	
Oral glucose-lowering drugs, n (%)	10 (18)
Insulin, n (%)	24 (42)
Lipid lowering drugs, n (%)	14 (26)
Antihypertensive drugs, n (%)	24 (42)

Data are mean ± SD. BMI; body mass index, eVFA; estimated visceral fat area, LDL-C; low density lipoprotein-cholesterol, HDL-C; high density lipoprotein-cholesterol, HOMA-IR; homeostasis model assessment of insulin resistance, IMT; imtima-media thickness.

Serum adiponectin concentrations correlated inversely with eVFA (Figure S1A) while CRP levels correlated positively with eVFA (Figure S1B). Insulin concentrations correlated significantly with eVFA (Figure S1C) and HOMA-IR tended to increase in parallel with increase in eVFA (Figure S1D). The leukocyte count, but not the erythrocyte count or platelet count, correlated significantly with eVFA (Figure S2A to S2C). Furthermore, the lymphocyte, monocyte, and neutrophil counts, but not those of eosinophils and basophils, correlated positively with eVFA (Figure S2D to S2H).

### Analysis of Gene Expression Profiles

Peripheral blood RNA samples from 28 subjects (BMI 31.9±6.0 kg/m^2^, VFA 199.4±89.4 cm^2^) were subjected to microarray analysis. The target probes were selected under the condition that significant signals were detected in more than 14 cases and thus 27969 genes were extracted for gene expression analysis. Table 2 lists the top 20 genes that correlated significantly with eVFA: 8 genes correlated positively and 12 genes correlated negatively with eVFA. Among these genes, the solute carrier family 46 member 3 (SLC46A3), which is classified as a membrane protein, showed the highest statistical significance with eVFA (P = 0.000006). Importantly, significant correlations with eVFA were also observed in genes related to oxidative stress and inflammation, such as peroxiredoxin 3 (PRDX3) (P = 0.00033), suppressor of cytokine signaling 3 (SOCS3) (P = 0.0007), and ORAI calcium release-activated calcium modulator 1 (ORAI1) (P = 0.0009). Interestingly, a negative correlation with eVFA was observed in period homolog 1 (PER1), which is classified as a transcription factor and recognized as a circadian clock gene (P = 0.0011).

**Table 2:** Correlation coefficients of peripheral blood cell gene expression with visceral fat adiposity.

Gene Symbol	Gene Name	P value
**Positively correlated genes**		
SLC46A3	solute carrier family 46, member 3	0.000006
DUSP3	dual specificity phosphatase 3	0.00007
DEF8	differentially expressed in FDCP 8 homolog	0.0002
APOM	apolipoprotein M	0.00033
PRDX3	peroxiredoxin 3	0.00033
SOCS3	suppressor of cytokine signaling 3	0.0007
LOC644538	hypothetical protein LOC644538	0.0007
DOK4	docking protein 4	0.0011
**Negatively correlated genes**		
TSGA14	testis specific, 14	0.00002
CABIN1	calcineurin binding protein 1	0.00007
ZFP36	zinc finger protein 36	0.0001
RAB37	RAB37, member RAS oncogene family	0.0002
PBXIP1	pre-B-cell leukemia homeobox interacting protein 1	0.00032
RABGAP1L	RAB GTPase activating protein 1-like	0.0004
SMPD1	sphingomyelin phosphodiesterase 1, acid lysosomal	0.0004
ZNF174	zinc finger protein 174	0.0006
C3orf16	chromosome 3 open reading frame 16	0.0006
CCND3	cyclin D3	0.0007
ORAI1	ORAI calcium release-activated calcium modulator 1	0.0009
PER1	period homolog 1	0.0011

**Figure 1:**
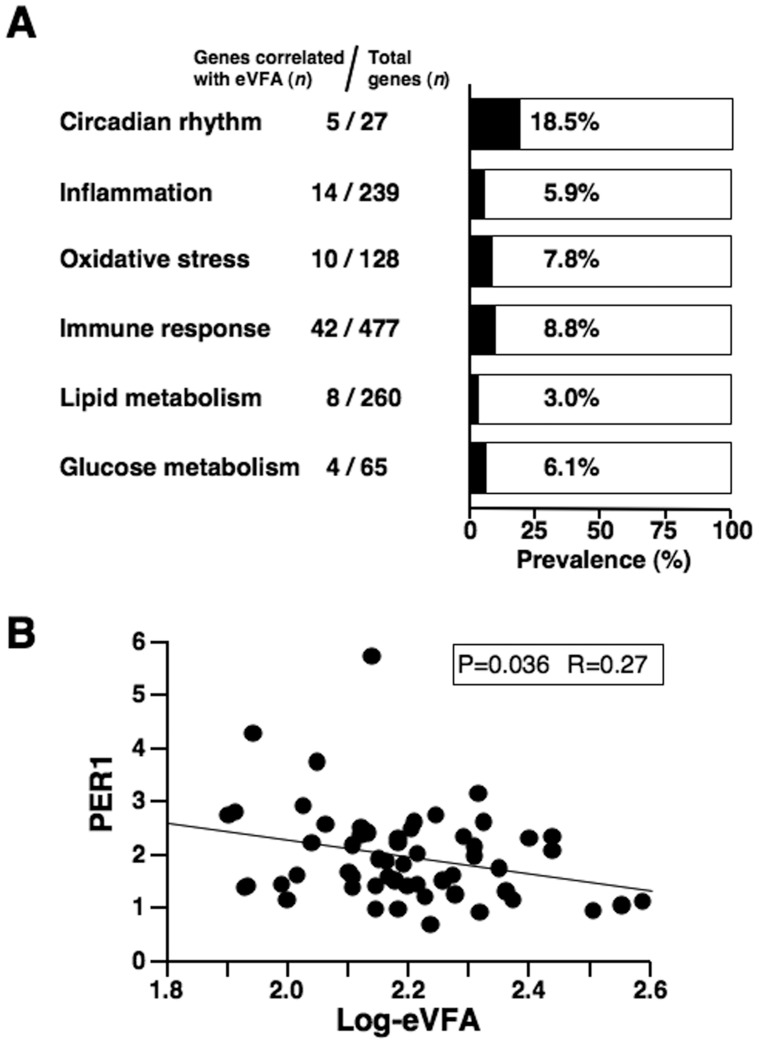
Gene expression profile in peripheral blood cells. (A) Prevalence of gene probes correlated with estimated visceral fat area (eVFA). Gene ontology analysis was performed based on the microarray data. (B) Correlation between PER1 mRNA level and eVFA. Total RNAs from peripheral blood cells of 57 subjects were subjected to RT-PCR.

Next, we conducted gene ontology (GO) analysis and searched for genes involved in circadian rhythm (GO:0007623), inflammation (GO:0006954), oxidative stress (GO:0006979), immune response (GO:0006955), lipid metabolism (GO:0006629), and glucose metabolism (GO:0006006). Figure 1A shows the prevalence of genes that showed significant correlation with eVFA. The number of circadian rhythm genes was small, but 5 genes (18.5%) showed significant correlation with eVFA. The frequencies of inflammation-, oxidative stress-, and immune response-related genes that correlated significantly with eVFA were 5.9%, 7.8%, and 8.8%, respectively. Furthermore, the frequencies of lipid metabolism- and glucose metabolism-related genes that correlated significantly with eVFA were 3.0% and 6.1%, respectively. Increasing evidence demonstrates a close relationship between the disturbance of circadian clock oscillator and the development of metabolic syndrome [12]–[14]. Table 3 shows gene probes related to circadian rhythm (GO:0007623). PER1, v-erb-b2 erythroblastic leukemia viral oncogene homolog 3 (ERBB3), clock homolog (CLOCK), prokineticin 2 (PROK2), and cryptochrome 2 (CRY2) correlated significantly with eVFA.

**Table 3:** Genes related to circadian rhythm.

Gene Symbol	Probe Name	P value
PER1	A_23_P89589	0.0011
ERBB3	A_23_P349416	0.0050
CLOCK	A_23_P419038	0.0120
PROK2	A_23_P97342	0.0230
CRY2	A_23_P127394	0.0489
CRY2	A_23_P388027	0.0565
CYP7B1	A_23_P169092	0.0669
CRY2	A_23_P158587	0.0699
AANAT	A_23_P152527	0.0704
CRY1	A_23_P36665	0.0864
PRF1	A_23_P1473	0.0939
HEBP1	A_23_P117082	0.0956
PHLPP1	A_23_P89762	0.1440
KCNMA1	A_23_P61150	0.1618
TIMELESS	A_23_P53276	0.2195
PER2	A_23_P411162	0.2494
PER2	A_23_P209320	0.3684
CRY1	A_24_P407235	0.4662
ATOH7	A_23_P378514	0.4755
ARNTL	A_24_P162037	0.5197
MAT2A	A_23_P401568	0.5893
NR1D1	A_23_P250227	0.7034
JUN	A_23_P420873	0.7405
HTR7	A_23_P500381	0.7585
MAT2A	A_32_P87703	0.9325
PROKR2	A_23_P412603	0.9702

PER1 : period homolog 1, ERBB3 : v-erb-b2 erythroblastic leukemia viral oncogene homolog 3, CLOCK : clock homolog, PROK2 : prokineticin 2, CRY2 : cryptochrome 2, CYP7B1 : cytochrome P450, family 7, subfamily B, polypeptide 1, ANNAT : arylalkylamine N-acetyltransferase, CRY1 : cryptochrome 1, PRF1 : perforin 1, HEBP1 : heme binding protein 1, PHPP1 : PH domain and leucine rich repeat protein phosphatase 1, KCNMA1 : potassium large conductance calcium-activated channel, subfamily M, alpha member 1, TIMELESS : timeless homolog, PER2 : Period homolog 2, ATOH7 : atonal homolog 7, ARNTL : aryl hydrocarbon receptor nuclear translocator-like, MAT2A : methionine adenosyltransferase II, alpha, NR1D1 : nuclear receptor subfamily 1, group D, member 1, JUN : jun oncogene, HTR7 : 5-hydroxytryptamine receptor 7, PROKR2 : prokineticin receptor 2.

### Association between PER1 and Metabolic Parameters

As shown in Figure 1A and Table 3, genes relating to circadian rhythm were highly correlated with eVFA. The highest correlation with eVFA was observed in PER1 among them. RT-PCR was, therefore, performed in 57 subjects to revalue the association of eVFA and PER1 mRNA levels in peripheral blood cells. As shown in Figure 1B, PER1 mRNA levels correlated negatively with eVFA (Figure 1B).

Table 4 lists the correlation coefficients for the relationship between PER1 and various metabolic parameters. Age- and sex-adjusted univariate analysis showed that PER1 correlated negatively with log-eVFA, HOMA-IR, WBC, and CRP. Stepwise multiple regression analysis revealed log-eVFA as a significant determinant of PER1.

**Table 4:** Correlation between PER1 and metabolic parameters.

	Univariate(non-adjusted)		Univariate(age,sex-adjusted)		Multivariate	
Parameter	r	p value	R	p value	p value	F value
Age	−0.28	0.031	–	–		
Sex	0.22	0.095	–	–		
BMI	−0.20	0.132	−0.27	0.047	–	–
Waist circumference (WC)	−0.26	0.044	−0.23	0.080		
Log-eVFA	−0.28	0.036	−0.29	0.023	0.005	8.969
Systolic blood pressure	−0.03	0.787	−0.03	0.786		
Diastolic blood pressure	0.05	0.705	−0.17	0.274		
Fasting glucose	−0.10	0.426	−0.12	0.354		
Hemoglobin A1c (JDS)	−0.17	0.206	−0.19	0.142		
HOMA-IR	−0.36	0.019	−0.42	0.013	0.090	3.074
AST	−0.13	0.329	−0.14	0.283		
ALT	−0.02	0.854	−0.10	0.439		
γ-GTP	−0.07	0.602	−0.08	0.551		
Total cholesterol	0.24	0.065	0.21	0.111		
LDL-C	0.19	0.149	0.17	0.202		
Triglyceride	0.10	0.456	−0.01	0.938		
HDL-C	0.11	0.389	0.20	0.128		
Creatinine	0.17	0.191	0.29	0.073		
Log adiponectin	−0.03	0.809	0.12	0.393		
WBC	−0.11	0.395	−0.37	0.011	0.087	3.128
CRP	−0.36	0.006	−0.37	0.003	0.096	2.968
Complication of DM		0.056	0.22	0.099		
Complication of HT		0.169	0.13	0.331		
Complication of DLP		0.788	0.02	0.885		
Mean IMT	0.02	0.886	0.10	0.551		

Data are mean ± SD. BMI; body mass index, eVFA; estimated visceral fat area, LDL-C; low density lipoprotein-cholesterol, HDL-C; high density lipoprotein-cholesterol, HOMA-IR; homeostasis model assessment of insulin resistance, DM; diabetes mellitus, HT; hypertension, DLP; dyslipidemia, IMT; imtima-media thickness.

## Discussion

The main findings of the present study were: *(1)* Visceral fat adiposity correlated with the expression of various genes related to circadian rhythm, inflammation, and oxidative stress, in peripheral blood cells. *(2)* Peripheral blood PER1 mRNA expression level correlated negatively with visceral fat area. *(3)* Visceral fat area was a significant determinant of PER1 mRNA level in peripheral blood cells.

Chronic low-grade inflammation is closely associated with the metabolic syndrome. Immune cell infiltration and production of reactive oxygen species (ROS) are increased in obese adipose tissue and such changes can cause adipocyte dysfunction. The latter can cause disorders of circulating fatty acids, ROS, and adipocytokines, which are located upstream in the development of metabolic syndrome and atherosclerosis [5]–[8], [15]–[17]. As shown in Table 2, several genes related to inflammation and ROS were associated with visceral fat adiposity, suggesting that inflammation of the adipose tissue may reflect on the expression of genes in peripheral blood cells. Interestingly, lymphocyte, monocyte, and neutrophil counts correlated positively with eVFA. The present data are in agreement with the reported increase in monocytes in obese subjects [18]. Such change in leukocyte subsets in visceral fat adiposity may be initiated by adipose local inflammation. Alternatively, it is also possible that the increase in the number of peripheral lymphocytes, monocytes, and neutrophils, which are somehow activated in bone marrow in visceral fat obesity, could results in the induction of local and/or systemic inflammation, with subsequent development of the metabolic syndrome. It is possible that some leukocyte subsets may affect the expression profile of certain genes, especially the mRNA level of PER1 in peripheral blood cells. PER1 mRNA level might be high in CD4-positive T cell rather than the other cells such as neutrophil, monocyte, CD8-positive T cell, and B cell, by analyzing microarray database (GSE22886)(data not shown), but further studies are needed to determine the exact leukocyte subtype(s) that influence peripheral blood PER1 mRNA level. In addition, target blood cell population of visceral fat should be identified in future.

Accumulating evidence indicates a close interrelationship between the circadian clock oscillator and metabolic syndrome [12]–[14]. Several genetic models of circadian disruption also exhibited metabolic disorders and vascular dysfunction [19]. One recent study highlighted the role of mouse Per genes in the development of obesity [20]. Furthermore, experimental evidence suggests that high-fat diet can alter the amplitude of peripheral circadian clock genes in mouse adipose tissue and liver [21]. In the present study, 18.5% of circadian genes in peripheral blood cells correlated significantly with eVFA (Figure 1A) and a significant correlation between PER1 mRNA level and eVFA was observed (Figure 1B). Other reports investigated circadian clock genes in human peripheral blood cells. In healthy male subjects, no distinct circadian changes were observed in the mRNA levels of PER2 and aryl hydrocarbon receptor nuclear translocator-like (ARNTL/BMAL1), whereas PER1 mRNA levels exhibited a clear oscillation during the 24-hour period with a peak expression level at 8 am [22]. We also obtained the preliminary data that the peripheral blood PER1 mRNA levels were oscillated with a peak expression level at 7∶30 am (data not shown). These data support the present findings that peripheral blood PER1 mRNA level was reduced in visceral fat accumulation since the blood samples were collected exactly at 7∶30 am in the present study. Circadian changes in Per1 mRNA were also reported in the mouse white adipose tissue [23] and disturbances of its expression were also reported in obese mice [24]. However, there is still a gap in our understanding of the circadian oscillation in mouse Per1 mRNA. Furthermore, the regulatory mechanism that control human PER1 expression in peripheral blood cells also remains uncertain. Haimovich et al [25] recently showed that a bolus administration of endotoxin resulted in down-regulation of PER1 mRNA in peripheral blood cells following a rise in plasma IL-6 and TNF-α levels but had no effect on melatonin secretory rhythm in human subjects [25]. Interestingly, our data (Table 4) showed that CRP was correlated with peripheral blood PER1 mRNA level. Considered collectively, it is possible that chronic low-grade inflammation could cause impairment of circadian oscillation of PER1 mRNA in peripheral blood cells with visceral fat accumulation. Alternatively, peripheral blood leukocytes with low PER1 mRNA level may have pro-inflammatory properties capable of initiating local inflammation in the adipose tissue. Further prospective studies are needed to examine whether dysregulation of circadian genes in peripheral blood cells can induce a vicious cycle, leading to the development of metabolic syndrome and cardiovascular events.

The present study has several limitations. Diabetes mellitus, dyslipidemia, and hypertension were common in the study population, since all subjects were inpatients. These metabolic diseases and medications could modulate the expression levels of various genes in peripheral blood cells directly or indirectly. The correlation between PER1 expression level and medication was also examined (data not shown), but there were no significant correlations in present study. Further studies will be needed in future to understand what kind of medications influence on peripheral blood cell mRNA expressions. In addition, the study participants were obese Japanese subjects (BMI ≥25 kg/m^2^) and visceral fat area was measured by BIA, not CT or MRI. Future studies are needed to analyze the gene expression profile in peripheral blood cells from not only obese subjects but also non-obese healthy (low VFA) subjects, although we obtained the preliminary data that peripheral blood PER1 mRNA levels were significantly higher in non-obese healthy volunteers than in the current study population (data not shown). The effects of diet- and exercise-induced visceral fat reduction on gene expression profile in peripheral blood cells should be investigated in future.

In perspective, gene expression profiling in peripheral blood cells may be applied to detect the function and condition of visceral fat tissues in human, although further studies are needed in future. These analyses may provide the new knowledge of metabolic syndrome and will achieve the novel diagnostic and therapeutic approaches for metabolic syndrome.

## Supporting Information

Figure S1 **Correlation between estimated visceral fat area (eVFA) and various blood parameters.** The homeostasis model−assessment of insulin resistance (HOMA-IR) was calculated as follows: HOMA-IR  =  fasting insulin (µU/mL)×fasting glucose (mg/dL)/405.
(TIFF)

Figure S2 **Correlations between estimated visceral fat area (eVFA) and peripheral blood cell count.**
(TIFF)
