# Supplementary figures and images for: A Pilot Investigation of Visceral Fat Adiposity and Gene Expression Profile in Peripheral Blood Cells
Source: PLoS One. 2012 Oct 16;7(10):e47377. doi: 10.1371/journal.pone.0047377 (PMC3472996; doi:10.1371/journal.pone.0047377)

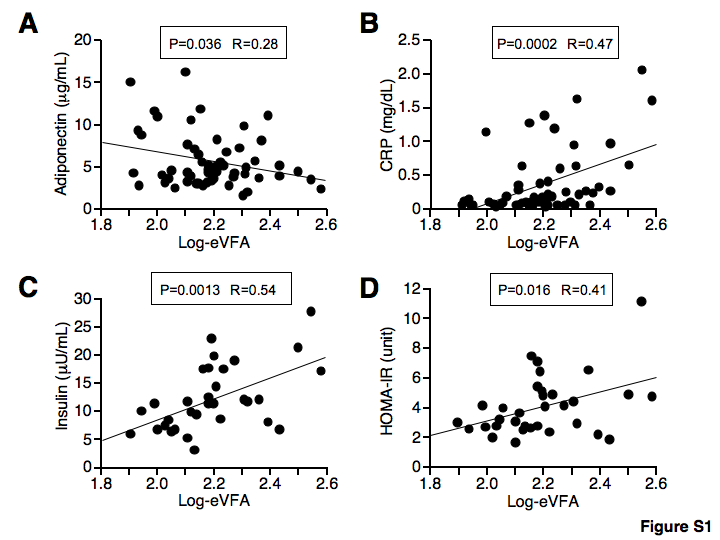

Supplement: Figure S1 — Correlation between estimated visceral fat area (eVFA) and various blood parameters. The homeostasis model−assessment of insulin resistance (HOMA-IR) was calculated as follows: HOMA-IR = fasting insulin (µU/mL)×fasting glucose (mg/dL)/405. (TIFF) [file pone.0047377.s001.tiff]

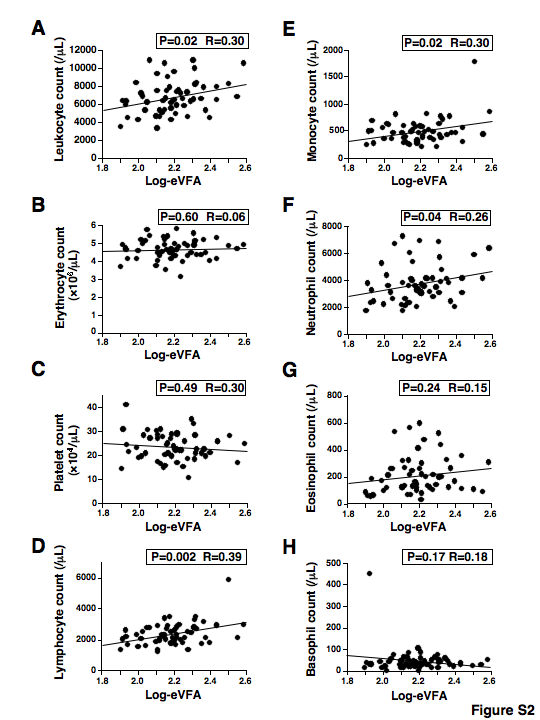

Supplement: Figure S2 — Correlations between estimated visceral fat area (eVFA) and peripheral blood cell count. (TIFF) [file pone.0047377.s002.tiff]
